# From Gene Knockouts to Genome Remodeling: Large DNA Fragment Deletion Technologies in Plants

**DOI:** 10.3390/plants15060909

**Published:** 2026-03-15

**Authors:** Jiayi Hou, Hui Li, Fengfeng Zhang, Dan Yang, Yan Xiong, Xiaoyue Zhu, Mingzhang Wen

**Affiliations:** 1 State Key Laboratory of Synthetic Biology, Frontiers Science Center for Synthetic Biology, Ministry of Education, School of Synthetic Biology and Biomanufacturing, Tianjin University, Tianjin 300072, China; monkey202111@126.com (J.H.);; 2 Haihe Laboratory of Sustainable Chemical Transformations, Tianjin 300192, China; 3 Fujian Provincial Key Laboratory of Haixia Applied Plant Systems Biology, Haixia Institute of Science and Technology and College of Life Sciences, Fujian Agriculture and Forestry University, Fuzhou 350002, China; 000q825126@fafu.edu.cn (H.L.);

**Keywords:** genome editing, CRISPR, prime editing, synthetic biology, plant biotechnology

## Abstract

Large DNA fragment deletion (LDFD) provides a powerful means to reconfigure plant genomes at the kilobase to megabase scale, enabling the dissection of genome function, elucidation of non-coding regulatory elements, modulation of gene dosage, reorganization of chromosomal architecture, and implementation of synthetic biology designs. In this review, we systematically compare the mechanisms, efficiencies, advantages, and limitations of the major LDFD technologies that have been applied in plants, including ZFNs, TALENs, CRISPR/Cas systems (Cas9, Cas12a, Cas3), site-specific recombinases, transposon-based systems, and prime editing-derived strategies. We highlight how plant-specific features of chromatin organization and DNA repair constrain large deletions, and discuss the current bottlenecks in achieving efficient, precise, and predictable LDFD across diverse crop genomes. Finally, we outline future directions for plant LDFD, emphasizing AI-assisted design of nucleases and recombinases, protein-directed evolution, and improved DNA- and RNP-based delivery systems. Together, these advances are expected to transform LDFD from a specialized tool into a broadly accessible platform for functional genomics, trait engineering and rational genome design in plants.

## 1. Introduction

**Figure 1:**
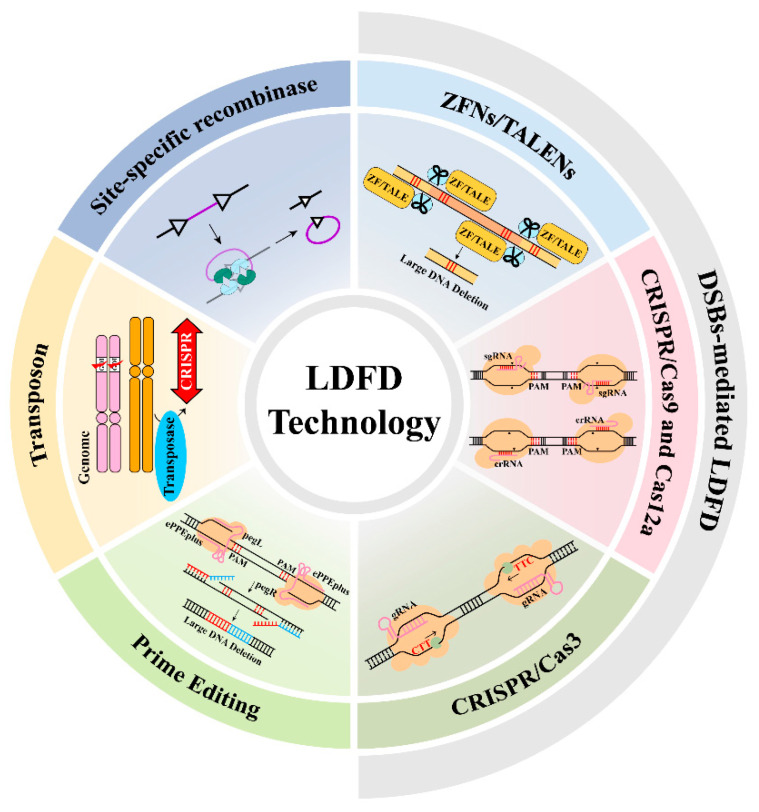
Overview of LDFD technologies.

Large DNA fragment deletion (LDFD) is defined as a type of genomic structural variation (SV) characterized by the precise removal of substantial, contiguous segments of the genome, occurring in both prokaryotic and eukaryotic organisms [1]. A widely used threshold for classifying such deletions as “large” is a deleted fragment size of ≥1 kb. Such large-scale deletions can significantly influence evolutionary trajectories, adaptive strategies, and pathogenicity. In vivo, LDFD can be induced by several mechanisms, including error-prone repair of DNA double-strand breaks (DSB), transposon mobilization, and non-homologous recombination (see Figure 1 for an overview LDFD technologies). Different species vary widely in their tolerance to these genetic alterations. Deletions that disrupt essential genes or key regulatory elements often result in lethality or severe developmental abnormalities. In contrast, the loss of redundant sequences or non-coding regions may enhance adaptive plasticity and environmental fitness, ultimately fostering evolutionary innovation. For instance, the rice mutant *spl39*, induced via heavy-ion beam irradiation, exhibits extensive genomic DNA deletions associated with increased bacterial blight resistance and elevated defense gene expression [2], whereas some bacterial pathogens acquire antibiotic resistance through adaptive genomic deletions of non-essential gene clusters [3].

The core value of studying LDFD lies in four interrelated aspects: (1) elucidating the regulatory logic of non-coding regions, particularly long-range regulatory elements such as enhancers and silencers; (2) dissecting how gene dosage imbalance shapes cellular and organismal phenotypes; (3) exploring the biological consequences of remodeling higher-order chromosomal architecture; (4) providing tools for genome streamlining and optimization in synthetic biology. By precisely engineering large deletions, scientists can mimic naturally occurring structural variants, construct disease and trait models, and rationally design more efficient synthetic biology systems.

With the rapid development of synthetic biology, LDFD technologies are playing increasingly important roles in plant research. In functional genomics, LDFD enables the removal of large regulatory regions, including distal non-coding elements, to assess their impact on gene expression and plant phenotypes, thereby elucidating gene function at both the locus and network levels. In crop improvement, tuning gene dosage via large deletions can modulate key agronomic traits such as yield, quality, and stress resistance [4,5,6]. In addition, LDFD provides a powerful tool to study how structural variants influence plant development and environmental adaptation, deepening our understanding of the relationship between genome structure, function and diversity, and providing new insights into plant evolutionary dynamics.

Currently, zinc finger nucleases (ZFNs) [7], transcription activator-like effector nucleases (TALENs) [8], and CRISPR/Cas technologies [9] are widely used to DSBs at defined positions flanking the target region. Repair of these DSBs relies on endogenous DNA repair pathways, primarily non-homologous end joining (NHEJ), homologous recombination (HR), and microhomology-mediated end joining (MMEJ), to generate LDFDs. Among these pathways, HR and MMEJ can mediate more precise deletions, while NHEJ usually results in error-prone repair [10]. Site-specific recombinases constitute another important class of tools for LDFD, they recognize specific recombination sites and excise the intervening sequences between co-oriented sites on the same chromosome. Recombinases can mediate efficient deletions without inducing DSBs or relying on endogenous DNA repair mechanisms [11]. However, recombinase-based LDFD requires prior insertion of recombination sites flanking the fragment of interest, and excision efficiency typically declines as the size of the targeted deletion increases [12].

In plant genome editing, the potential and limitations of different LDFD technologies have attracted increasing attention. Although ZFNs, TALENs, CRISPR/Cas9, and CRISPR/Cas12a (also known as Cpf1) can all induce large deletions, they are still used predominantly for targeted gene knockouts [13,14,15]. Recombinases and transposons, which enable precise excision of specific DNA segments, are more widely applied to remove selectable markers and residual editing cassettes after transformation [16,17]. More recently, DualPE, a prime editor-derived dual prime editing RNA (pegRNA) strategy, and the CRISPR/Cas3 system have emerged as promising platforms for programmable LDFD in plants [18,19]. However, important challenges remain: DualPE often suffers from low editing efficiency, whereas the CRISPR/Cas3 system can generate highly heterogeneous deletion products.

**Figure 2:**
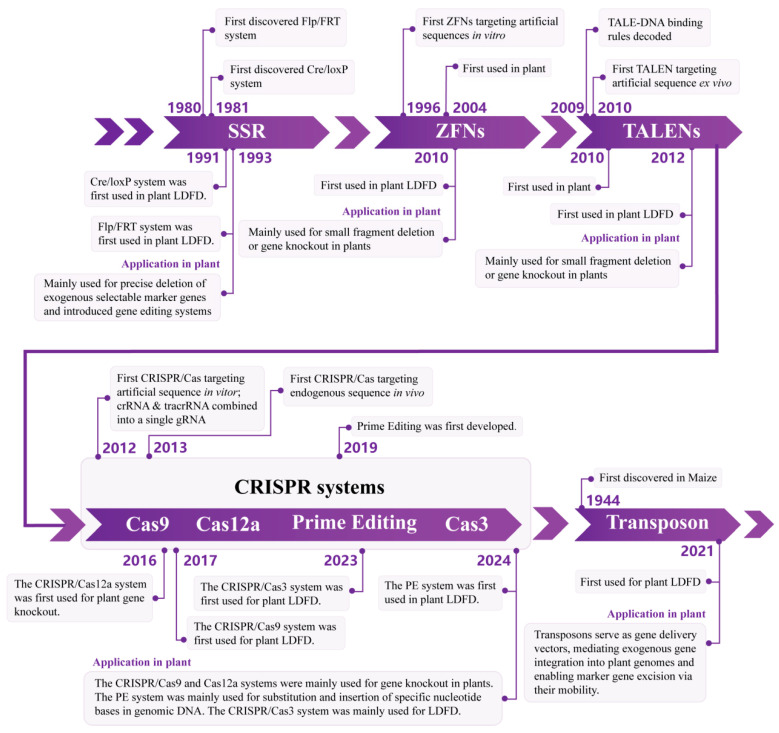
Timeline of LDFD technologies.

In this review, we systematically summarize DNA deletion tools currently applied in plants, highlighting their mechanisms, efficiencies, advantages, and limitations in achieving LDFD (Table 1). In addition, a timeline of LDFD technologies is illustrated in Figure 2. We further discuss how protein-directed evolution and related engineering strategies can be leveraged to optimize these systems and expand their applicability. By providing an integrated overview of existing technologies and future directions, we aim to offer a conceptual and technical framework for advancing LDFD as a versatile platform for plant functional genomics, trait engineering and synthetic genome design.

## 2. LDFD Based on DSBs

**Figure 3:**
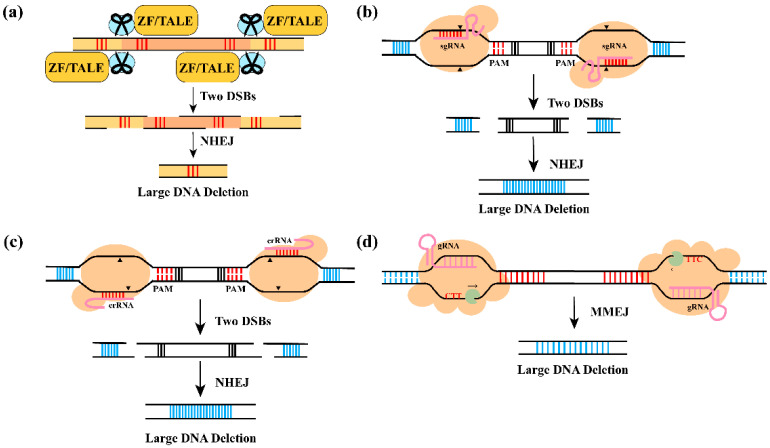
**Mechanism of DSBs-mediated LDFD**. (**a**) ZFNs and TALENs: These customized nucleases generate two DSBs at specific loci, enabling LDFD via the NHEJ repair pathway. (**b**,**c**) CRISPR/Cas9 and CRISPR/Cas12a: A single DSB induced by Cas9 or Cas12a causes small indels via NHEJ, whereas two DSBs followed by NHEJ repair result in LDFD (The black triangle indicates the cleavage site of Cas protein). (**d**) CRISPR/Cas3: The Cascade complex recognizes target DNA via crRNA and recruits Cas3 (green part). Cas3 progressively degrades DNA through iterative cleavage, generating LDFDs.

Gene-editing technologies can be classified into three categories based on the function of sequence-specific nucleases: ZFNs, TALENs, and CRISPR-derived systems. These tools can all introduce DSBs at specific genomic loci. ZFNs and TALENs are constructed by fusing a customizable DNA-binding domain to the non-specific FokI endonuclease domain; dimerization of the FokI domain enables cleavage and DSB formation at the target loci (Figure 3a) [13]. Among these platforms, TALENs often show higher activity and are more amenable to high-throughput assembly than ZFNs [25].

The CRISPR/Cas system represents an adaptive immune mechanism that defends prokaryotes against invading genetic elements, including bacteriophages and plasmids. The core components of CRISPR technology consist of two key elements: CRISPR-associated Cas genes and CRISPR arrays [26]. Based on their structural organization and sequence features, CRISPR/Cas systems are further divided into three major types. Types I and III systems require the assembly of large multi-Cas protein complexes for function, whereas Type II systems rely on a single Cas9 protein to accomplish targeted DNA cleavage [27]. The CRISPR/Cas9 system generates DSB three-base pairs upstream of the protospacer adjacent motif (PAM), typically the 5′-NGG-3′ sequence (Figure 3b) [28]. Conventional CRISPR/Cas9 systems use a Pol III promoter to express a chimeric sgRNA that recruits Cas9 to the desired genomic locus. This architecture has been adapted for multi-gene knockouts by expressing tandem or polycistronic sgRNA cassettes, including those processed by tRNA or Csy4-based systems, thereby enabling simultaneous editing of multiple sites [29,30]. Cas12a differs from Cas9 in several critical aspects and can functionally complement the CRISPR/Cas9 system. It recognizes T-rich PAM sequences, most frequently TTTN, thereby targeting genomic regions inaccessible to NGG-dependent Cas9 [31]. Moreover, Cas12a cleaves the distal region of the protospacer in a staggered manner, producing cohesive 4–5 nt 5′ overhangs (Figure 3c) [32]. Unlike Cas9, which requires an approximately 80 nt tracrRNA, Cas12a depends only on a short crRNA without the need for additional tracrRNA, thus simplifying the guide RNA architecture and shortening the expression cassette [33]. In addition, the Type I CRISPR/Cas3 system has been widely employed for LDFD and is characterized by the synergistic action of the Cascade surveillance complex and the Cas3 helicase-nuclease. In contrast to the relatively localized DSBs induced by CRISPR/Cas9 and Cas12a, Cas3 mediates multi-step, unidirectional processing that drives extensive degradation of contiguous DNA (Figure 3d) [34]. In terms of design, compared with ZFNs and TALENs, the CRISPR/Cas genome editing platform is more economical and technically accessible, since its target specificity is encoded by a short RNA rather than engineered protein domains. Design principles and screening software tools for programmable nucleases and gRNAs in plant genomes are summarized in Table 2. Compared with protein-based nucleases, RNA-guided target recognition generally enables higher throughput target design, lower cost, and reduced cytotoxicity.

Simultaneous induction of DSBs at two target sites within the same gene or chromosome enables targeted deletion of specific genomic fragments via the NHEJ, HR, and MMEJ repair pathways [8,11]. The NHEJ pathway is active throughout the cell cycle and directly ligates broken DNA ends without requiring homologous sequences [35]. HR is more active in late S/G2 phase and requires vectors carrying 0.5–1 kb homology arms. Although it confers high editing fidelity, its application is limited by complicated vector construction, cell cycle dependency, and cell-type specificity, especially in systems with low recombination efficiency [36]. MMEJ depends on short microhomologous sequences (MHs) of 4–25 bp and can mediate site-specific integration of exogenous fragments; this repair mechanism is active from M phase to early S phase and, in some context, can yield higher insertion efficiency than HR-based strategies [10]. However, the MMEJ repair pathway necessitates the identification of appropriate MHSs flanking the fragment to be deleted and the design of editing targets adjacent to these sequences, which restricts its practical application. Furthermore, studies in model organisms including plants have demonstrated that MMEJ frequently leads to sequence deletions between pairs of MHs near the DSBs introduced by genome editing [37,38].

Typically, LDFD efficiencies achieved in plants using the DSB-based technologies described range from 1% to 5%, with the notable exception of CRISPR/Cas3 [10,19,20,21,22,23]. The Dvu I-C system, developed from the CRISPR/Cas3 platform, exhibits exceptional genome-editing activity, achieving efficiencies exceeding 80% for long-fragment deletion (>1 kb) in maize and rice. By employing paired crRNAs, the Dvu I-C system can efficiently introduce controllable LDFDs with sizes reaching at least 20 kb [19]. Cas3-mediated LDFD exhibits superior efficiency, stemming from fundamental differences in its mechanism of action and its compatibility with DNA repair pathways. Cas3 possesses both helicase and HD nuclease activities. Following targeting by the Cascade complex, Cas3 degrades DNA unidirectionally and processively along the targeted DNA strand. Moreover, the PAM-in configuration of paired crRNAs enables bidirectional degradation, making it inherently suited for LDFD. Additionally, the DNA ends with MHs generated by Cas3 degradation are more compatible with MMEJ. In contrast, other strategies predominantly rely on NHEJ, which tends to produce small insertions and deletions(indels) and shows drastically reduced efficiency for long-fragment deletion.

Although DSB repair-mediated technologies enable LDFDs in plant genomes, they suffer from substantial limitations, including overall low editing efficiency and frequent formation of unintended byproducts at DSB ligation junctions, which markedly compromise the fidelity and reliability of genome modification. Accordingly, developing novel strategies that circumvent DSB induction and enable precise editing of large genomic segments has become a critical direction for overcoming current technical bottlenecks.

## 3. LDFD Based on Site-Specific Recombinases

Site-specific recombinases (SSRs) catalyze highly efficient and precise DNA rearrangement (integration, excision, and inversion) by recognizing short DNA motifs and assembling a synaptic complex in which a catalytic tyrosine or serine residue executes nucleophilic attack on the DNA backbone, forming and resolving transient covalent intermediates. Based on the active site residue, SSRs are classified into the tyrosine and serine recombinase families [39]. Tyrosine recombinases are further subdivided according to whether they act on identical or distinct recognition sites. Recombinases utilizing identical sites generally mediate reversible reactions, whereas those recognizing distinct sites typically mediate irreversible events. Serine recombinases are categorized by molecular size into large (~60 kDa) and small (~23 kDa) subfamilies. Despite recognizing identical sites, small serine recombinases typically catalyze conservative excision or integration between short inverted or direct repeats. In contrast, large serine recombinases catalyze a broader repertoire of reactions, including integration, excision, and inversion [40]. Widely used SSRs include Causes Recombinase (Cre) from bacteriophage P1 and Flipase (Flp) from *S. cerevisiae* [40,41].

**Figure 4:**
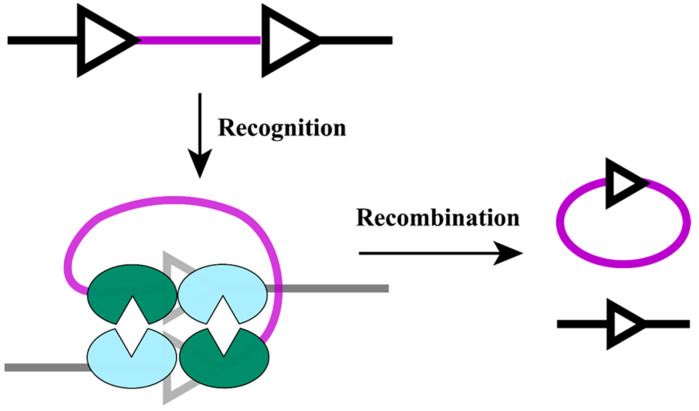
**Mechanism of site-specific recombinase-mediated LDFD**. By recognizing and binding to recognition sites, SSRs mediate precise cleavage and ligation of DNA fragments between sites, thereby achieving precise LDFDs. Triangular symbols represent recombinase recognition sites; blue and green parts denote recombinase monomers, each recognizing a half-site and assembling into a functional tetrameric complex; the purple segment indicates the fragment to be deleted. This process enables precise cleavage and ligation of DNA fragments between sites, facilitating efficient site-specific recombination.

LDFDs have been achieved predominantly using tyrosine recombinases, which enable the precise, scar-minimal deletion of large genomic segments in plants (Figure 4) [10,24,42,43]. Notably, the newly developed programmable chromosome engineering (PCE) system enables precise excision by inserting LoxAR2 and Lox71 recombination sites (designed to suppress Cre-mediated reverse recombination) at both ends of the target deletion fragment via a dual-pegRNA strategy, followed by recombination catalyzed by AI-engineered hyperactive Cre recombinases (e.g., cm24, with a 3.5-fold higher recombination efficiency than the wild-type enzyme). This system excises DNA fragments ranging from 800 kb to 4 Mb, with a maximum deletion efficiency of 1.0% in rice [24]. The growing toolkit of orthogonal recombinase–target pairs has also enabled the construction of synthetic gene circuits that integrate multiple independent excision and inversion events to reprogram gene expression patterns during development or in response to environmental cues [12,44]. In many of these systems, recombinase expression is driven by inducible systems, i.e., blue light-, galactose/estradiol-, and dexamethasone (DEX)-inducible systems, allowing precise temporal control of LDFD [12,45,46].

Selectable marker genes are routinely used to identify transgenic events, but are often unnecessary after initial selection. SSRs have been used primarily to excise marker cassettes rather than to generate locus-specific knockouts. Strategic placement of recognition sites flanking marker or cargo sequences allows precise excision once stable transformants are obtained, thereby producing clean, marker-free lines. Split recombinase systems further enhance biosafety and flexibility. For instance, N-Cre and C-Cre fragments can be expressed in separate parental lines. Functional Cre is reconstituted only in F1 hybrids, allowing an efficient one-generation excision and avoiding multi-generational segregation [42]. A Visual Monitoring DNA-Free Multigene Editing System (VMDFGE) couples reporter expression to Flp activity at composite LoxP::FRT sites, enabling real-time monitoring of excision events. Upon induction, Flp removes foreign DNA with minimal genomic footprint, thereby reducing off-target effects and enhancing transgene biosafety [47].

Despite their attractive precision, SSRs exhibit several limitations when used for LDFD in plants. A negative correlation exists between the excision efficacy of recombinase enzymes and the size of the targeted DNA segment. In addition, recombinase activity in plant cells is usually lower than in microorganisms, likely reflecting differences in chromatin organization, DNA methylation, and the nuclear environment, which can impair target recognition and catalytic turnover [43,48]. The high sequence specificity of SSRs, while minimizing off-target events, also limits the number of naturally available target sites in a given genome. Two complementary strategies are being employed to overcome these bottlenecks through rational engineering and computational design of recombinases with altered or broadened specificity. (a) in silico mining of microbial genomic datasets to identify and characterize site-specific recombinases; and (b) deep-learning frameworks, such as RecGen, which learn sequence–activity relationships from large training sets and generate tyrosine recombinase variants de novo for user-defined genomic targets [49,50].

Recent advances in recombinase engineering have yielded powerful platforms that enable large and programmable deletions. Dual designer recombinase-induced genome deletion (dDRiGD) uses pairs of engineered tyrosine recombinases to flank and precisely excise specific human genomic loci. Co-expression of two such recombinases enables the removal of a ~1.4 kb DNA fragment with an efficiency of approximately 30% [51]. The spacer sequence within loxP sites is a major determinant of recombination efficiency. RecS3, a recombinase with altered substrate specificity, was isolated by screening a library of 6000 loxP variants. RecS3 recognizes distinct spacer variants and exhibits substantially enhanced excision efficiency, underscoring the potential of directed evolution to expand the scope of recombinase-based technologies [52]. Furthermore, fusion of recombinases with programmable DNA-binding domains can boost both activity and specificity at challenging loci. For instance, N- or C-terminal fusion of zinc finger domains to recombinases at engineered lox-zif sites increased recombination efficiency by up to ten fold, providing a valuable strategy for retargeting SSRs to otherwise inaccessible regions [53].

## 4. LDFD Based on Prime Editing

The Prime Editing (PE) system is a novel genome editing technology that consists of two core components: a fusion protein of an RNA-programmable nickase (nCas9) and a reverse transcriptase (RT), and a prime editing guide RNA (pegRNA) [54]. Unlike conventional CRISPR gRNAs, the pegRNA contains three functional elements: a gRNA that specifies the genomic target, a primer binding site (PBS), and a reverse transcriptase template (RTT) encoding the desired edit. Guided by the pegRNA, the nCas9-RT fusion protein binds the target site and nicks the non-target strand. The exposed 3’ end then hybridizes with the PBS, enabling the RT to synthesize new DNA that incorporates the desired sequence [55].

The PE system has been optimized through multiple generations (PE1-PE7) to improve efficiency and functionality [56,57,58,59,60]. While PE can, in principle, introduce arbitrary base substitutions and small insertions/deletions (generally <30 bp), most applications to date have focused on short-fragment editing [18]. Efforts to improve prime editing efficiency and scope include both protein and RNA engineering. On the protein side, nCas9 has been optimized, for example by substituting SpyCas9 (H840A) with FnCas9 to broaden the targetable range and reduce off-target activity [61]. In reverse transcriptase engineering, David Liu’ s team generated novel reverse transcriptase variants via phage-assisted continuous evolution and structure-guided rational engineering, developing the PE6a–d prime editors [59]. PE6c harbors an evolved and engineered Tf1 retrotransposon-derived reverse transcriptase and shows significantly higher editing efficiency in rice than other variants, making PEc and its derivatives promising optimal tools for monocot prime editing [62]. On the RNA side, the 3’ extension of pegRNAs has been engineered with stability-promoting motifs such as pseudoknots (evopreQ1, mpknot), G-quadruplexes, exonuclease-resistant xrRNA, or stem-loop aptamers (MS2, PP7, Csy4, BoxB) to increase editing efficiency [63,64,65,66,67]. Additional protein engineering strategies, such as optimizing nuclear localization signals, introducing beneficial point mutations, fusing functional peptides or protein domains, and inhibiting DNA mismatch repair via dominant-negative MLH1 (MLH1dn), further increase the efficiency and robustness of PE [58,68,69].

**Figure 5:**
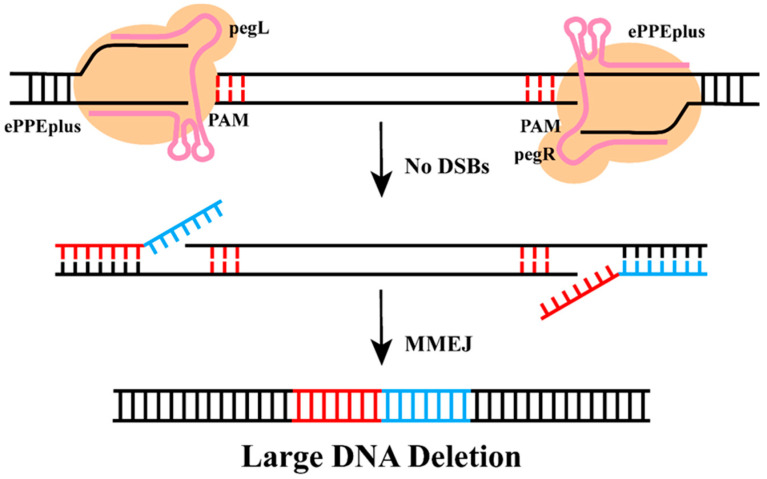
**Mechanism of LDFD based on PE.** PE directly rewrites DNA sequences via reverse transcriptase and pegRNA, enabling precise deletion of specific fragments. The dual-pegRNA strategy utilizes paired pegRNAs to direct the targeted excision of target fragments, enabling directional deletion of discrete genomic regions. Red and blue segments represent microhomology sequences (MHS) that cooperate via the MMEJ pathway to enable efficient and precise LDFD.

Building on these advances, a number of PE-derived methodologies have been developed specifically to enable programmable DNA deletions in mammalian cells [70,71,72,73,74,75]. The DualPE system, which combines the plant-optimized editor ePPEplus with dual-engineered epegRNAs, has achieved heritable LDFDs of ~365.9 kb in wheat, with a deletion efficiency that is notably independent of fragment length (Figure 5) [18]. With the exception of EXPERT, most of these methods rely on dual pegRNAs for complementary-strand targeting. Systems like PRIME-Del, twinPE, and GRAND combine nCas9-RT with two PAM-in pegRNAs to facilitate kb-scale DNA deletions, replacements, or insertions [70,72,73]. By contrast, PEDAR and PETI utilize a wild-type Cas9-RT fusion together with dual pegRNAs, enabling not only 1–10 kb DNA deletions but also Mb-scale DNA inversions or translocations [71,74]. However, since Cas9-RT induces DSBs, the editing precision of PEDAR and PETI is constrained.

Overall, PE-based LDFD platforms exhibit higher precision and, in many cases, superior efficiency compared with conventional DSB-based approaches. As prime editors, pegRNA architectures, and plant-optimized delivery strategies continue to be improved, PE-derived systems are expected to support increasingly larger and more accurate deletions, making them a powerful and versatile toolkit for programmable large-scale genome remodeling in plants.

## 5. LDFD Based on Transposons

Transposons constitute a major genomic component in plants, representing approximately 15–85% of their genome [76]. Based on their transposition mechanisms, transposons are categorized into two major classes: Class I retrotransposons and Class II DNA transposons [77]. Class II DNA transposons translocate via a “cut-and-paste” mechanism, which enables their widespread application in plants for selectable marker excision [18]. In these systems, a transposase catalyzes precise excision from the donor site, stabilizes the excised transposon DNA, and mediates reintegration of the excised DNA into a new genomic locus [78].

**Figure 6:**
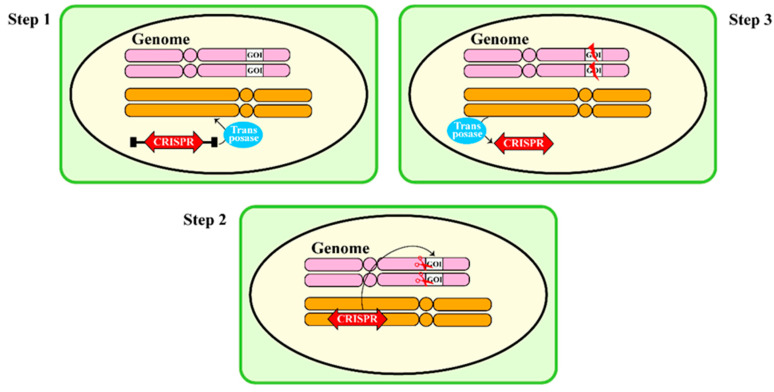
**Mechanism of LDFD based on transposon**. Class II DNA transposons complete transposition within the genome through the cut-and-paste mechanism: the transposase (colored in blue) catalyzes precise excision of the transposon from the genome. The CRISPR expression cassette (colored in red) guides the system to the target site, enabling accurate integration into a new genomic target.

The *piggyBac* transposon, a Class II system originally isolated from *Trichoplusia ni*, has been validated in plant systems [79]. In rice, *piggyBac* has been integrated with CRISPR/Cas9 to enable precise gene editing followed by clean excision of the transposon cargo, leaving only the intended genomic modification (Figure 6) [17]. This approach is particularly valuable for asexually propagated crops, where classical genetic segregation cannot be used to remove transgenes. However, *piggyBac* transposition activity remains suboptimal in some plant species [80]. Furthermore, a Transposon-Mediated, Ultra-Clean Selectable Transformant (TRUST) system has been developed based on the endogenous Ac/Ds transposon system [81]. By integrating transposon elements with fluorescent protein and anthocyanin biosynthesis genes, a visual selection system was established for generating ultra-clean transgenic maize containing only the target expression cassette, achieving an average transformation of 15.5%.

Plant transposon systems enable multidimensional applications in biotechnology. For targeted insertion, fusion of a transposase to Cas9 or Cas12a facilitates precise DNA integration and targeted gene modulation [78]. For LDFD, Class II DNA transposons utilize their “cut-and-paste” mechanism to excise integrated genomic fragments. However, this approach requires pre-existing transposon insertions and carries a risk of fragment re-integration. In addition, the performance of transposon systems in plants is constrained by epigenetic regulatory networks. DNA methylation can strongly suppress transposon activity through chromatin remodeling and transcriptional regulation [82]. Despite these challenges, transposon-based systems remain indispensable in totipotent regeneration platforms such as callus and suspension cultures, providing a robust foundation for precise genome engineering that minimizes or eliminates residual exogenous sequences.

## 6. Comparison and Selection of LDFD Technology Systems

In plant LDFD, DSB-dependent strategies (ZFNs, TALENs, and CRISPR/Cas) exhibit distinct technical features and application niches. ZFNs and TALENs mediate targeted LDFDs via site-specific cleavage but are hampered by cumbersome vector assembly, high experimental complexity, elevated costs, modest efficiency, and suboptimal precision—factors impeding their scalable applications in plants. By contrast, CRISPR/Cas has become the cornerstone of plant genome editing, leveraging facile gRNA design and flexible programmability to enable efficient, site-specific deletions with simplified workflows. Successfully deployed for polyploid genome engineering and multiplex gene knockout in rice, wheat, and maize, this system demonstrates exceptional versatility for modifying complex genomes with multicopy genes, highly homologous sequences, and large genome sizes [83,84,85]. Notably, CRISPR/Cas3, with its unidirectional processive nuclease activity, enables kilobase-scale deletions and offers greater capacity and potential efficiency than Class 2 effectors (Cas9, Cas12a). However, its **multicomponent Cascade**-Cas3 architecture requires further optimization for vector construction and in planta use [86].

Conventional DSB-dependent gene knockouts and LDFD rely on endogenous plant DNA repair pathways, leading to low editing fidelity, unintended edits, and genomic instability. In contrast, DSB-independent tools (site-specific recombinases, prime editing, transposon-mediated systems) circumvent DSB-associated damage and therefore hold substantial promise for precision deletions. Among these, PE enables one-step precise deletion via rationally designed dual pegRNAs, with heritable edits stably transmitted in rice and wheat; enhanced variants (PE5max, PE6) further boost efficiency [58,59]. Recombinase- and transposon-mediated LDFD, however, require pre-engineered genomic modifications: recombinases depend on flanking cognate recognition sites, whereas transposons require pre-installed inverted repeats (IRs) for precise excision. Additionally, recombinase efficiency correlates negatively with target length (declining progressively with fragment size) and recombination activity in plant cells is considerably lower than in microbes. Transposon-mediated LDFD also carries re-integration risks, potentially inducing undesired genetic alterations [12,17,49].

For delivery systems, major plant approaches (Agrobacterium-mediated transformation, biolistic bombardment, direct protein delivery, and viral vectors) present distinct trade-offs. Agrobacterium-mediated transformation is widely adopted but is limited by labor-intensive tissue culture and genotype dependency [87]. Biolistic delivery enables vector-free editing but may cause genomic lesions [88]. Protein and viral delivery support transgene-free editing but restrict cargo molecular size and complexity [89,90]. ZFNs’ compact size allows direct protein delivery, while TALENs, Cas effectors, and Cascade-Cas3 remain inefficient for this route due to their larger size and intricate architecture [86,91]. Notably, engineered RNA viral vectors (TSWV, SYNV, and BSMV) enable stable delivery of large modules such as Cas9, Cas12a, and base editors [90]. TSWV operates in solanaceous crops (tobacco, tomato, and pepper), SYNV has been reported to yield >90% heritable editing efficiency in tobacco, and BSMV generates germline-transmissible mutations in wheat, greatly expanding the application range of viral delivery systems.

Rational selection of LDFD tools depends on target size, genomic context, and intended application. (1) For small deletions (1–30 bp) and gene knockout, CRISPR/Cas9 and CRISPR/Cas12a are preferred because they are well-established and compatible with multiplex gRNA designs [92]. (2) For medium-to-large deletions (5–100 kb), CRISPR/Cas3 often leads in deletion capacity and efficiency due to its processive nuclease activity [19]. (3) For transgene-free editing, recombinases enable scarless excision of transgenes and selectable markers, particularly valuable in non-model or asexually propagated crops. While viral vectors can support delivery of large cargos, agrobacterium-mediated transient expression and biolistic RNP delivery also achieve transgene-free editing [42,47,93,94]. (4) For ultra-large deletions (>100 kb), PE-derived strategies can offer higher precision for programmable deletions and suggest a route to improve DSB-dependent LDFD accuracy by flanking target regions with MHs to redirect repair toward MMEJ [18].

## 7. Directed Evolution, Deep Learning, and AI-Assisted Protein Design for LDFD

With the growing demand for customized genome editors including recombinases and polymerases, protein engineering has become central to advancing LDFD technologies. Directed evolution provides a powerful framework for tailoring these proteins by iteratively diversifying sequences and selecting improved variants [95]. Directed evolution typically employs three primary strategies: random mutagenesis, rational mutagenesis, and recombination-based approaches such as gene shuffling [96].

For proteins and polypeptides lacking sequence–structure–function information, random mutagenesis remains an essential route to access beneficial sequence variants. Error-prone PCR (epPCR) generates large, targeted mutant libraries in vitro. Following high-throughput functional screening, variants with desired properties, such as enhanced activity, altered specificity, or improved stability, can be isolated and subsequently identified [97]. By directly coupling the recombination capacity of the recombinase to bacterial survival, a recombinase-capacity-coupled survival selection system was established in *Escherichia coli*. Stepwise increases in selection pressure enabled directional enrichment of hyperactive recombinase variants capable of recognizing shortened recombination sites. After seven rounds of iterative screening, the recombinase recognition sites attP/attB were successfully truncated from 46/44 bp to 28 bp [98].

Conventional epPCR typically requires multiple rounds of vector construction, library transformation and screening, making the process labor-intensive and time-consuming. To address this, continuous in vivo directed evolution platforms have been developed that decouple mutagenesis from host genome replication. Orthogonal replication systems employ an engineered error-prone DNA polymerase (e.g., TPDNAP1-4-2) dedicated to the replication of a specialized plasmid, enabling targeted mutagenesis of genes of interest at mutation rates on the order of ~10^−6^, much higher than endogenous genomic mutation rates (10^−10^–10^−9^) [99]. This strategy has also been extensively applied to the directed evolution of enzymes and functional proteins tailored for plant systems. Based on the OrthoRep technology, the prokaryotic thiazole synthase MhTHI4 was successfully evolved into a highly active mutant adapted to the mild aerobic and low-sulfide intracellular environment of plant cells [100]. Furthermore, phage-assisted continuous evolution (PACE) is widely utilized to optimize the performance of gene-editing tools. This system couples the evolution process to the phage life cycle by linking *gIII* activity to target gene expression, in which mutagenic plasmids introduce mutations into the target gene and selection pressure is applied to enable efficient enrichment of beneficial variants [101]. The IntePACE system, developed on the basis of PACE, significantly improves serine integrase activity, with multi-gene integration efficiency reaching up to 80% [102]. Meanwhile, PACE has generated nuclease variants with broadened PAM recognition profiled (e.g., SlugCas9-NNG, evoCjCas9, and SpCas9), as well as novel reverse transcriptase variants used to develop the PE6a–d series of prime editors [59,103,104,105]. Notably, PE6c displays significantly higher editing efficiency in rice than other variants, making it a promising tool for prime editing in monocots [62]. In the field of high-throughput screening in plant systems, Gao’s group has recently achieved a major breakthrough by establishing geminivirus replicon-assisted in planta directed evolution (GRAPE) [106]. This technique engineers geminiviruses into regulatable DNA replicons and couples gene function to rolling-circle replication (RCR), thereby converting viral DNA replication into a rapid and quantifiable readout for protein activity. Using this system, researchers can screen approximately 10^5^ mutants on a single *Nicotiana benthamiana* leaf in just four days. Together, gene-editing tools improved via directed evolution have been successfully applied in plants, and these advances make it feasible to rapidly screen and validate additional emerging gene-editing tools in planta.

In parallel, deep learning has emerged as a transformative approach for protein engineering. Built on deep neural networks and large-scale sequence and structural datasets, modern deep learning models can learn high-dimensional representations of protein sequences, predict properties such as stability, activity, and substrate specificity, and generate new sequences with desired attributes [107,108,109]. RecGen, a deep learning-driven generative algorithm for recombinase engineering, efficiently predicts functional recombinases capable of targeting novel DNA recognition sites [49]. Such models can dramatically narrow the experimental search space by enriching libraries with high-value candidates before wet-lab screening. Nonetheless, the widespread application of deep learning–based protein engineering is currently limited by the need for extensive training data, substantial computational resources, and specialized bioinformatics expertise.

Recent advances in foundation models and generative protein design tools offer new opportunities to integrate artificial intelligence directly into enzyme engineering for LDFD. AI-guided continuous evolution pipelines can iteratively propose beneficial mutations, evaluate them in silico, and feed prioritized variants into high-throughput experimental screens. For example, AI-assisted design has been used to optimize base editors and polymerases, yielding variants with narrowed editing windows, enhanced fidelity, or improved organelle-targeted activity, thereby boosting the efficiency and specificity of genome editing systems [110]. Furthermore, the Cre recombinase mutant cm24 evolved via the AiCE_rec_ approach exhibits 3.5-fold higher recombination efficiency than the wild type and has been successfully applied to LDFD in plants [24]. In parallel, AI-based tools such as RFdiffusion and AlphaFold3 enable de novo generation and structure-guided refinement of proteins and protein complexes, including inhibitors targeting DNA repair factors such as MLH1. Together, these tools can be used to rationally design accessory proteins that suppress mismatch repair and thereby substantially enhance prime editing efficiency and compatibility in diverse cellular contexts [111].

Overall, the deep integration of directed evolution, continuous in vivo evolution platforms, and AI-driven protein design is poised to accelerate the development of next-generation LDFD tools for plants. Iterative cycles of model-guided library design, high-throughput functional screening, and continuous evolution can be used to engineer recombinases with tailored recognition sites, CRISPR nucleases with optimized processivity and specificity, and prime editors with improved efficiency and safety. As these approaches are increasingly adapted to plant systems and combined with plant-optimized expression and delivery strategies, they are expected to overcome current bottlenecks in efficiency, precision, and species dependence, ultimately enabling routine, scalable LDFD for plant functional genomics, trait improvement, and synthetic genome design.

## 8. Conclusions and Future Perspective

In plant systems, LDFD platforms based on ZFNs, TALENs, CRISPR/Cas, site-specific recombinases, prime editing, and transposons each exhibit distinct strengths and limitations. Looking ahead, genome editing technologies are poised for continued refinement to address current bottlenecks in LDFD. Future efforts will focus on developing systems with higher deletion efficiency across kilobase-megabase scales, improved control over deletion boundaries, expanded targeting scope in complex plant genomes, and more versatile DNA-, RNA-, and protein-based delivery strategies tailored to diverse crop species. At the same time, optimizing editing precision and safety in complex eukaryotic genomes will be crucial for enabling routine use of LDFD in functional genomics and trait engineering. As these advances converge, LDFD technologies are expected to evolve from specialized tools into broadly accessible platforms that accelerate both basic plant biology and the rational design of improved crop varieties.

## Figures and Tables

**Table 1:** Features of Technologies for Large DNA Fragment Deletion in Plants.

Technology	Editor	DSB Repair Pathway	Editing Size	Efficiency	Precision	Advantages	Limitations	Application in Plants
ZFNs and TALENs	ZFNs	NHEJ	Gene cluster deletion: Up to 55 kb Large chromosomal segment deletion: Up to 9 Mb (rare)	Moderate	Imprecise	High specificity: more precise Protein-DNA recognition (vs. CRISPR), lower off-target rate, applicable to high-GC/recurrent sequences	Complex design and unstable efficiency across different target sequences	Arabidopsis [20]
	TALENs	NHEJ	>6 kb	Moderate	Imprecise			Arabidopsis, tobacco [21]
CRISPR/Cas9 and CRISPR/Cas12a	CRISPR/Cas9	NHEJ	Up to 304 kb	Relatively High	Imprecise	High flexibility: virtually universal genomic site targeting; high-throughput applicability: gRNA library-based large-scale screening; multi-fragment deletion: simultaneous LDFD multiplex gRNAs	Possessing off-target and chromosome rearrangement risks	Wheat [22]
		MMEJ	Up to 20.7 kb	Relatively High	Relatively precise			Rice [10]
	CRISPR/Cas12a	NHEJ	Up to 1 Mb	Relatively high	Imprecise			Soybean [23]
CRISPR/Cas3	Dvu I-C system	NHEJ	Up to 20 kb	High	Imprecise	Outperforms CRISPR/Cas9 and CRISPR/Cas12a in efficiency of large chromosomal deletions, versatility, and tolerance to repetitive sequences	Imprecise Cas3-mediated LDFDs (unpredictable length, boundaries); complex vector construction; chromosomal rearrangement risk	Maize, Rice [19]
Site-specific recombinase	PCE/RePCE	No DSB	Up to 4 Mb	Very high	Precise	Enables efficient and precise deletion, superior to conventional NHEJ with no extra mutations or chromosomal aberrations	Requires pre-insertion of recombinase sites, hindering one-step deletion of site-specific DNA fragments.	Rice [24]
Prime Editing	DualPE	No DSB	Up to 2 Mb	High	precise	No DSBs (reduced genome injury risk); high precision (suitable for clinical use)	Applicable only to small (<100 bp) deletions; large-fragment efficiency needs optimization	Wheat, tobacco and tomato [18]
Transposon	A *piggyBac*-mediated transgenesis system	No DSB	~9.8 kb deletion covering the entire CRISPR/Cas9 expression cassette	High	Precise	Precisely excises redundant sequences (maintains genome purity); tailored for asexually propagated crops (addresses non-segregable transgene removal); compatible with low-efficiency genome editing tools	Deletion relies on prior transposase integration; potential re-integration risk	Rice [17]

**Table 2:** Resources for programmable nucleases design and gRNA design in CRISPR/Cas system.

	Software	Source
**ZFNs**	Addgene	https://www.addgene.org/ (accessed on 10 March 2026).
	Zinc Finger Targeter	http://www.zincfingers.org/software-tools.htm (accessed on 10 March 2026).
	ZFNGenome	http://www.zincfingers.org/ (accessed on 10 March 2026).
**TALENs**	Addgene	https://www.addgene.org/ (accessed on 10 March 2026).
	TAL Effector Nucleotide Targeter 2.0 (TALE-NT)	https://tale-nt.cac.cornell.edu/ (accessed on 10 March 2026).
	E-TALEN 2.5	http://www.e-talen.org/ (accessed on 10 March 2026).
	CHOPCHOP 3.0.0	http://chopchop.cbu.uib.no/ (accessed on 10 March 2026).
**sgRNA in CRISPR/Cas9 system**	Addgene	https://www.addgene.org/crispr/ (accessed on 10 March 2026).
	GPP sgRNA Designer	https://portals.broadinstitute.org/gpp/public/analysis-tools/sgrna-design (accessed on 10 March 2026).
	CHOPCHOP 3.0.0	http://chopchop.cbu.uib.no/ (accessed on 10 March 2026).
	Synthego Design Tool	https://design.synthego.com (accessed on 10 March 2026).
	CRISPick	https://portals.broadinstitute.org/gppx/crispick/public (accessed on 10 March 2026).
	CRISPOR 5.2	http://crispor.tefor.net/ (accessed on 10 March 2026).
	Cas-OFFinder 2.4	http://www.rgenome.net/cas-offinder/ (accessed on 10 March 2026).
	CCTop-CRISPR/Cas9	https://cctop.cos.uni-heidelberg.de/ (accessed on 10 March 2026).
	CRISPR Guide RNA Design tool	https://www.benchling.com/crispr/ (accessed on 10 March 2026).
	Cas-Designer	http://www.rgenome.net/cas-designer/ (accessed on 10 March 2026).
	Cas-Analyzer	http://www.rgenome.net/cas-analyzer/ (accessed on 10 March 2026).
	CRISPR-PLANT v2	https://www.genome.arizona.edu/crispr2/ (accessed on 10 March 2026).
	CRISPRseek 3.22	https://bio.tools/crisprseek (accessed on 10 March 2026).
	ATUM	https://www.atum.bio/eCommerce/cas9/ (accessed on 10 March 2026).
	E-CRISP 5.4	https://www.e-crisp.org/ (accessed on 10 March 2026).
	RGEN Tools	http://www.rgenome.net/ (accessed on 10 March 2026).
	sgRNAcas9-AI	http://123.57.239.141:8080/ (accessed on 10 March 2026).
	CRISPR-P 2.0	http://crispr.hzau.edu.cn/CRISPR2/ (accessed on 10 March 2026).
	AGEseq	https://galaxyproject.org/use/ageseq-aspendb/ (accessed on 10 March 2026).
	Stupar Lab’s CRISPR Design	https://stuparlab.cfans.umn.edu/protocols/crisprcas9-glycine-max (accessed on 10 March 2026).
**crRNA in CRISPR/Cas12a system**	CHOPCHOP 3.0.0	http://chopchop.cbu.uib.no/ (accessed on 10 March 2026).
	CRISPOR 5.2	http://crispor.tefor.net/ (accessed on 10 March 2026).
	PE-Analyzer	http://www.rgenome.net/pe-analyzer/#! (accessed on 10 March 2026).
**pegRNA in Prime Editing system**	pegFinder	http://pegfinder.sidichenlab.org/ (accessed on 10 March 2026).
	PE-designer	http://www.rgenome.net/pe-designer/ (accessed on 10 March 2026).

## Data Availability

The original contributions presented in this study are included in the article. Further inquiries can be directed to the corresponding authors.
